# Inhibitory Effect of Arctigenin from Fructus Arctii Extract on Melanin Synthesis via Repression of Tyrosinase Expression

**DOI:** 10.1155/2013/965312

**Published:** 2013-05-27

**Authors:** Hwayong Park, Kwang Hoon Song, Pil Mun Jung, Ji-Eun Kim, Hyunju Ro, Mi Yoon Kim, Jin Yeul Ma

**Affiliations:** ^1^KM-Based Herbal Drug Research Group, Korea Institute of Oriental Medicine, Yuseong-Daero 1672, Yuseong-Gu, Daejeon 305-811, Republic of Korea; ^2^Department of Biological Sciences, College of Biosciences and Biotechnology, Chungnam National University, Daehak-Ro 99, Yuseong-Gu, Daejeon 305-764, Republic of Korea; ^3^Department of Dermatology, School of Medicine, Chungnam National University, Munhwa-Ro 266, Daejeon 301-747, Republic of Korea

## Abstract

To identify the active compound arctigenin in Fructus Arctii (dried seed of medicinal plant *Arctium lappa*) and to elucidate the inhibitory mechanism in melanogenesis, we analyzed melanin content and tyrosinase activity on B16BL6 murine melanoma and melan-A cell cultures. Water extracts of Fructus Arctii were shown to inhibit tyrosinase activity in vitro and melanin content in **α**-melanocyte stimulating hormone-stimulated cells to similar levels as the well-known kojic acid and arbutin, respectively. The active compound arctigenin of Fructus Arctii displayed little or no cytotoxicity at all concentrations examined and decreased the relative melanin content and tyrosinase activity in a dose-dependent manner. Melanogenic inhibitory activity was also identified in vivo with zebrafish embryo. To determine the mechanism of inhibition, the effects of arctigenin on tyrosinase gene expression and tyrosinase promoter activity were examined. Also in addition, in the signaling cascade, arctigenin dose dependently decreased the cAMP level and promoted the phosphorylation of extracellular signal-regulated kinase. This result suggests that arctigenin downregulates cAMP and the tyrosinase enzyme through its gene promoter and subsequently upregulates extracellular signal-regulated kinase activity by increasing phosphorylation in the melanogenesis signaling pathway, which leads to a lower melanin content.

## 1. Introduction

Researches on natural products, including traditional medicine and herbs, have been increasing recently due to an increase in demand for complementary and alternative medicines with less side effect and increased safety [1]. In dermatological research, many studies have been performed to determine the mechanisms behind the regulation of melanogenesis and to identify clinically useful hypopigmenting agents [2, 3]. Agents from medicinal herbs and natural resources have been reported in many previous studies [4, 5]. In addition to dermatological research, many other fields have shown increased interest in identifying agents from natural resources, including cosmetics, functional foods, beverages, and feed additives in livestock [6–9]. In the cosmetic field, many studies have attempted to identify medicinal herbs and traditional medicines that can inhibit melanin and tyrosinase activity, which could be used as skin-whitening agent [10]. Many types of natural resources and products including various plants (whole plant or part) and extracts of various solvents have been screened with this goal in mind [11, 12]. In regard to skin, great efforts to develop materials that inhibit melanin biosynthesis and/or tyrosinase have been made for the development of skin-whitening agents [13].

Melanin exists elsewhere in nature and is found in almost all of the living organisms, including humans, where the high melanin content is found in skin, hair, and eyes [14]. It is now well known as a key determinant of color and tone of each tissue, which is dependent on the amount and distribution of melanin. Melanin is synthesized through a complex biochemical process, that is, melanogenesis, which typically occurs in melanosomes of epidermal melanocyte [15]. In this process, tyrosinase converts tyrosine to 3,4-dihydroxyphenylalanine (DOPA) through hydroxylation, which is then oxidized into dopaquinone (4-(2-carboxy-2-aminoethyl)-1,2-benzoquinone) and indole-5,6-quinone. This compound then leads to the synthesis of melanin. Tyrosinase functions as a hydroxylase and oxidase and can convert tyrosine into DOPA and can convert DOPA into dopaquinone [16, 17]. Melanogenesis is stimulated by UV irradiation and *α*-melanocyte stimulating hormone (*α*-MSH), which increase tyrosinase expression and melanin content by binding to melanocortin 1 receptor (MC1R). Activated MC1R and chemical compound forskolin and isobutylmethylxanthine (IBMX) also stimulate melanogenesis by activating cAMP in the melanocyte-specific pathway, and cAMP can in turn increase melanogenic signaling enzymes through protein kinase A (PKA), which induces microphthalmia-associated transcription factor (MITF) expression [18–20].

Among medicinal herbs, Fructus Arctii (FA) has been traditionally used for the treatment of inflammatory sore throats, urticaria, and furuncle. Arctiin and arctigenin (ATG) in *Arctium lappa* (AL) have been shown to inhibit leukemic B-cell hybridoma proliferation, human keratinocyte cell growth, and allergies, improve aging skin, and have ameliorative effects on experimental glomerulonephritis [21–25]. Also, these compounds have been reported to be novel cytotoxic and cancer chemopreventive agents in the medicinal herb bardanae fructus and *Saussurea medusa* [26, 27]. Arctiin and ATG were identified as the major compounds in *Centaurea sphaerocephala* ssp. *Polyacantha* and were also found in the AL used in this study [28, 29].

In this study, we investigated the inhibitory effect of FA extract (FAE) and ATG on tyrosinase and melanin synthesis and identified melanogenic inhibitory effect in both cell lines of B16BL6 and melan-A and also in zebrafish embryo. We found that these extracts downregulate cAMP, tyrosinase enzyme, and its gene promoter and promote extracellular signal-regulated kinase (ERK) phosphorylation in murine melanoma B16BL6 cell cultures to levels that were comparable with kojic acid (KA) and arbutin, which are widely used melanogenesis inhibitors.

## 2. Materials and Methods

### 2.1. Chemicals and Reagents

Arbutin, *α*-MSH, DOPA, dimethyl sulfoxide (DMSO), dithiothreitol (DTT), KA, mushroom tyrosinase, 3-(4,5-dimethylthiazol-2-yl)-2,5-diphenyltetrazolium bromide (MTT), Triton X-100, TRI reagent, Tween 20, and phenylthiourea (PTU) were purchased from Sigma-Aldrich (St. Louis, MO, USA). Lipofectamine 2000 reagent was obtained from Life Technologies (Carlsbad, CA, USA), and Dual Luciferase Assay System was from Promega (Madison, WI, USA). Antibodies against ERK, phosphorylated-ERK (p-ERK), and tyrosinase were obtained from Cell Signaling Technology (Danver, MA, USA) and Abcam (Cambridge, UK), respectively.

### 2.2. Preparation of Herb Extract

Medicinal herb was obtained from local vendors in Daejeon, Korea. FA (50 g) was immersed in 1 L of water for 1 h at room temperature to enhance the extract yield and boiled for 3 h. The extract was then filtered through 106 *μ*m test sieve (Retsch, Germany), freeze dried, and stored at −20°C until used. In the experiments, the extract was dissolved in Dulbecco's phosphate buffered saline (DPBS) at a concentration of 20 mg/mL, centrifuged (13,000 rpm, 10 min, 4°C), and the acquired supernatant was filtered through 0.2 *μ*m syringe filter.

### 2.3. Cell Culture and Viability Assay

Murine melanoma cell line B16BL6 and non-neoplastic cell line melan-A were maintained in Dulbecco's Modified Eagle Medium (DMEM, Lonza, USA) and RPMI 1640, respectively, supplemented with 10% (v/v) fetal bovine serum and 1x penicillin-streptomycin mixture (Gibco, USA) in a humidified 5% CO_2_ incubator at 37°C. In the cell viability assay, cells were seeded on 96-well plate in 100 *μ*L of culture media (1.5 × 10^4^ cells/well). The cells were further incubated for 24 h with or without ATG. The viability of cultured cells was determined by the reduction of 3-(4,5-dimethylthiazol-2-yl)-2,5-diphenyl tetrazolium bromide to formazan, as described previously with slight modifications [30]. In brief, 10 *μ*L of the MTT solution (5 mg/mL) was added to each well and incubated for 1 h at 37°C. Following incubation, the medium was removed, cells were washed with DPBS, and 100 *μ*L of DMSO was added to dissolve formazan precipitated by reduced MTT. The absorbance at 570 nm of the negative control (without inhibitor) was measured and the percentage of inhibition was calculated as follows: inhibition (%) = [OD 570 nm (ATG)/OD 570 nm (control)] × 100.

### 2.4. In Vitro Tyrosinase Inhibition Assay

Tyrosinase inhibition was determined spectrophotometrically as described previously, with minor modifications [31]. To examine the effect of FAE on tyrosinase activity in vitro, 50 *μ*L of DOPA (20 mM), KA (60 *μ*g/mL), FAE, and 100 *μ*L of tyrosinase (250 U/mL) were mixed together in 96-well plate and incubated at 37°C for 10 min. The absorbance was then measured using a microplate reader at 475 nm. DOPA, KA, and tyrosinase were dissolved in 0.1 M potassium phosphate buffer (pH 6.6) at the concentrations indicated above. Concentrations and volumes of each reagent were determined based on preliminary optimization experiments performed in our laboratory (data not shown). For control, cells were treated with DOPA, potassium phosphate buffer and tyrosinase, with no tyrosinase inhibitor. All the experiments were carried in triplicate.

### 2.5. Measurement of Melanin Contents

The melanin content was measured as described previously [32]. B16BL6 murine melanoma cells and melan-A cells (each 5 × 10^4^ cells/dish) were seeded on 6-well plate and treated with arbutin (positive control) or ATG. Melanogenesis stimulator *α*-MSH (100 nM) was used to induce melanin synthesis in both cell lines. After 72 h, cells were harvested, washed with DPBS, and collected. Cell pellets were then completely lysed with 1 N NaOH at 60°C for 1 h, and the absorbance at 405 nm of 200 *μ*L of the lysates was measured using microplate reader. The protein concentrations and melanin contents for each treatment were determined using the BCA protein assay kit (Thermo Scientific, USA) and were compared with the negative control (without arbutin and inhibitor).

### 2.6. Cellular Tyrosinase Activity Assay

Tyrosinase activity was measured as described previously, with minor modifications [33]. In brief, cells were seeded in 60 mm culture dish at a density of 1 × 10^5^ cells/dish and incubated for 24 h. After serum starvation for 24 h, the cells were further incubated for 72 h with or without ATG. Cells were harvested, washed in DPBS, and lysed with lysis buffer (1% Triton X-100 in 0.1 M sodium phosphate buffer pH 6.8, containing protease inhibitor) and subjected to a freeze-thaw cycle (−80°C 30 min/25°C 25 min/37°C 5 min). After centrifugation (13,000 rpm, 10 min), protein concentrations were determined using the BCA protein assay kit and the concentrations were adjusted to 200 *μ*g/mL. Cell lysate supernatants (each 50 *μ*L) were transferred to 96-well plate and mixed with 100 *μ*L of DOPA (20 mM) and 50 *μ*L of DPBS. After incubation at 37°C overnight, the absorbance at 475 nm was measured using a microplate reader. Cellular tyrosinase activities were determined as the ratio of tyrosinase content over total protein content (ng/*μ*g).

### 2.7. RNA Isolation and Quantitative RT-PCR

B16BL6 cells were treated with ATG for 1 h and further incubated for 24 h with *α*-MSH treatment. Total RNA was isolated using TRI reagent and 1 *μ*g of each samples was reverse-transcribed using AccuPower RT PreMix (Bioneer, Korea) according to the manufacturer's instructions, and aliquots of cDNA were subjected to real-time quantitative polymerase chain reaction (RT-qPCR) with AccuPower 2X Greenstar qPCR Master Mix (Bioneer, Korea). A forward primer sequence of 5′-GGCCAGCTTTCAGGCAGAGGT-3′ and reverse primer sequence of 5′-TGGTGCTTCATGGGCAAAATC-3′ were used for tyrosinase exon 1. Thermal cycling of PCR amplification was carried out as follows: initial denaturation at 95°C for 10 min, followed by 40 cycles of denaturation at 95°C for 10 s, annealing at 60°C for 30 s, and elongation at 72°C for 30 s using Eco Real-Time PCR System (Illumina, USA). For **β**-actin, forward primer 5′-TGTCCACCTTCCAGCAGATGT-3′ and reverse primer 5′-AGCTCAGTAACAGTCCGCCTAGA-3′ were used in the same thermal cycling conditions as described for the tyrosinase PCR experiments.

### 2.8. Construction of Mouse Tyrosinase Promoter Reporter

Upstream region, including the tyrosinase promoter, was amplified and isolated from genomic DNA by PCR using the following primers: forward 5′-GGTACCTTCAACCCTTTTCTATGTCC-3′ (−2267 to −2242) and reverse 5′-CTCGAGCACAGACTTCTTTTCCAGCAAC-3′ (−9 to +19). Genomic DNA information was obtained from http://genome.ucsc.edu/. The underlined sequences of each primer were not present in the genomic DNA and were artificially attached to confer restriction enzyme recognition sites for DNA cloning. The PCR product (2,286 bp) was then introduced into the T-Blunt vector system. The inserted PCR product was digested with restriction enzyme *Xho* I and *Kpn* I and then subsequently cloned into the same enzyme site of the pGL3-basic vector, which contained a luciferase coding sequence in its upstream region. Cloned DNA was identified by sequencing and enzyme digestion followed by sizing on the agarose gel electrophoresis.

### 2.9. Transfection and Luciferase Assay

For the analysis of CRE (cAMP response element) promoter activity, murine melanoma B16BL6 cells in 24-well dishes were transfected with 0.2 *μ*g of CRE-Luc vector and 0.05 *μ*g of Rlu, using 2 *μ*g of lipofectamine 2000 reagent in a total reaction volume of 200 *μ*L. After 24 h, the transfection medium was changed and the cells were treated with ATG for 1 h and further incubated with *α*-MSH for 24 h at 37°C. Cells were then washed with DPBS and lysed with Triton X-100 (1% in 25 mM Tris-phosphate buffer, pH 7.8) containing ethylenediaminetetraacetic acid (EDTA, 2 mM) and DTT (2 mM). Luciferase activity was determined using a Dual-Luciferase Assay system. All reagents were prepared as described in the manufacturer's instruction, and the data are represented as the ratio of firefly to Renilla luciferase activity (Fluc/Rluc).

### 2.10. Western Blot Analysis

Cells were treated with ATG or arbutin for 1 h, further incubated for 24 h at 37°C with *α*-MSH, and lysed with Triton X-100 (1% in 50 mM sodium phosphate buffer pH 6.8, containing protease inhibitor). The proteins in the cell lysate were separated by 10% sodium dodecyl sulfate- (SDS-) polyacrylamide gel electrophoresis and blotted onto a nitrocellulose membrane. The blotted membrane was incubated for 1 h with 5% nonfat milk in Tris-buffered saline (pH 7.6) containing 0.05% Tween 20. Subsequently, the membrane was incubated overnight with the anti-goat tyrosinase antibody (1 : 1000), and further incubated for 1 h with the anti-goat antibody conjugated with horseradish peroxidase (1 : 10,000). For ERK, the membrane was incubated overnight with ERK and p-ERK antibody (1 : 1000), and then incubated for 1 h with the anti-rabbit antibody conjugated with horseradish peroxidase (1 : 10,000). The *β*-actin antibody (1 : 1,000) was used for normalization. The signals were visualized using the enhanced chemiluminescence reagent (ECL western blotting substrate, Thermo Scientific, USA).

### 2.11. Observation of Depigmenting Effect in Zebrafish

Inhibitory effect of ATG on melanin pigmentation was observed in vivo using zebrafish embryo following the previous report with slight modification [34]. Zebrafish embryos were obtained from natural spawning of wild type AB*. The embryos were raised in standard fish water at 28.5°C. ATGs were dissolved in DMSO and added directly in fish water at the final concentration of 1, 10, and 100 *μ*M. As control, DMSO or PTU (well-known melanogenic inhibitor) treated embryos were analyzed together with the embryos of ATG treated. Live embryos at 40 hpf (hour post-fertilization) were classified and taken pictures.

## 3. Results and Discussion

### 3.1. Inhibitory Effect of FAE on In Vitro Tyrosinase Activity and *α*-MSH-Mediated Melanogenesis in B16BL6 Cells

**Figure 1:**
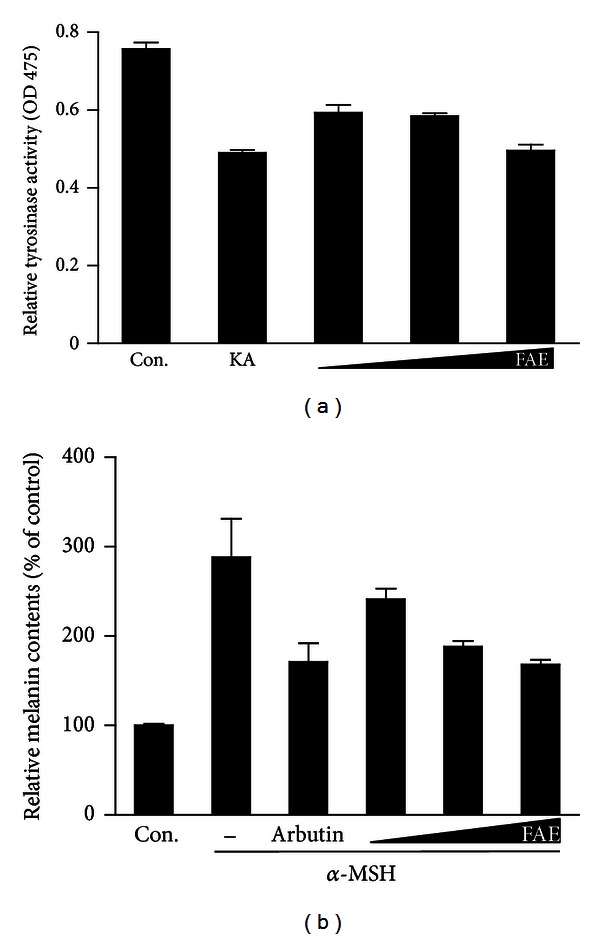
The inhibitory effect of FAE on tyrosinase activity in vitro (a) and melanin content in murine B16BL6 cells stimulated with 100 nM *α*-MSH (b). Cells were treated with KA (60 **μ**g/mL) or arbutin (100 **μ**g/mL) and FAE at different doses (1, 100, 500 **μ**g/mL). In vitro tyrosinase activities were measured based on the absorbance at 475 nm, and melanin contents are depicted as a percentage (%) of control. Each value was expressed with the mean ± SD of three independent experiments.

*Arctium lappa* (common name Burdock) has been used as a therapeutic herb in Asia, Europe, and North and South America for hundreds of years. It has also long been cultivated and used as a dietary and nutritional food, especially in Asia [35]. Here, we investigated the effect of FAE on tyrosinase activity and melanin contents using an in vitro screening system. These results were also compared with positive controls, KA, and arbutin. FAE was shown to inhibit tyrosinase activity in a dose-dependent manner (Figure 1(a)). We also examined the effect of FAE on cellular melanin synthesis (Figure 1(b)). Consistent with tyrosinase inhibition, melanin synthesis was also significantly inhibited by FAE in a dose-dependent manner. These results indicate that FAE has a significant inhibitory effect against not only tyrosinase activity but also cellular melanin synthesis, showing a similar activity as the positive control KA and arbutin. This suggests that FAE might be a useful natural compound for inhibition of tyrosinase activity and melanin synthesis.

### 3.2. Effect of ATG on the Cytotoxicity of B16BL6 Cells

**Figure 2:**
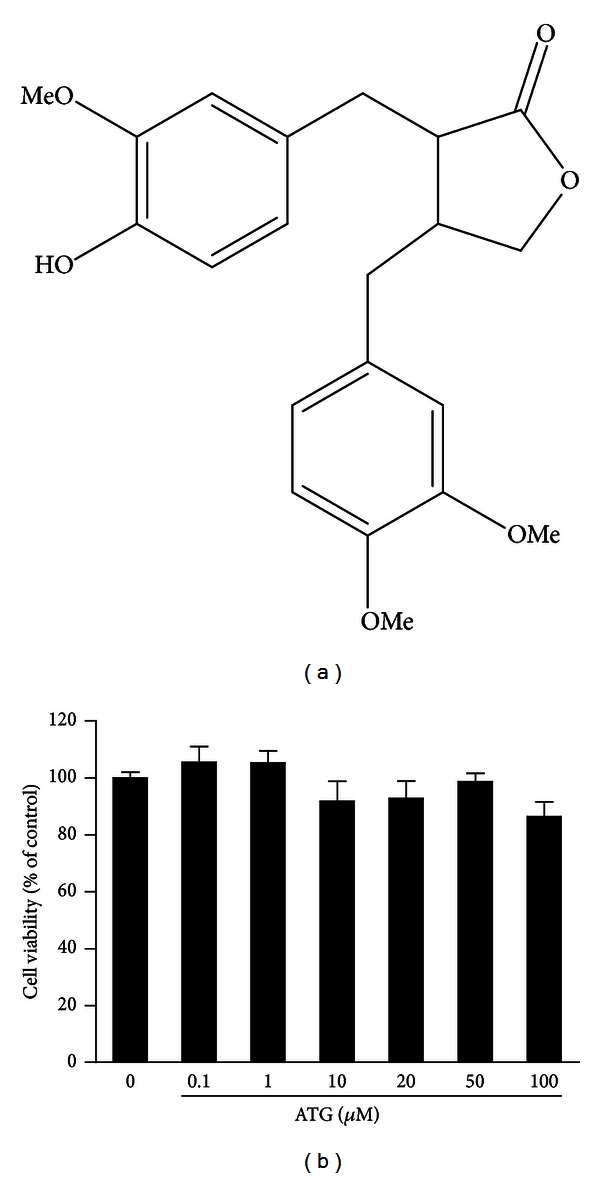
Chemical structure (a) and cytotoxicity of ATG at different treatment doses (b). Values of cytotoxicity were represented as a percentage (%) of control, with mean ± SD of three independent experiments.

Arctiin and ATG (Figure 2(a)), the main compounds of AL, have been reported to contain a variety of biological activities [36]. Among those compounds, we selected ATG because it has been shown to have beneficial biopharmaceutical properties and activities in the treatment of many diseases [37, 38]. However, the potential of using ATG for dermatological applications has not been examined until now, except one study that examined the photoprotective effect of fermented FA in terms of its effects on decreasing the expression of matrix metalloproteinase 1 (MMP-1) mRNA [39]. To investigate whether the ATG activity is related to the inhibitory effect on tyrosinase and melanin synthesis, we analyzed cellular tyrosinase activity and melanin contents in vitro. The cytotoxicity of ATG was also examined and we found that there was no significant cytotoxicity at concentrations up to 100 *μ*M (Figure 2(b)). This is consistent with other studies, which reported that ATG did not show any cytotoxic effect against B16BL6 cells at concentrations up to 100 *μ*M [40].

### 3.3. Inhibitory Effect of ATG on Melanin Content and Tyrosinase Activity

**Figure 3:**
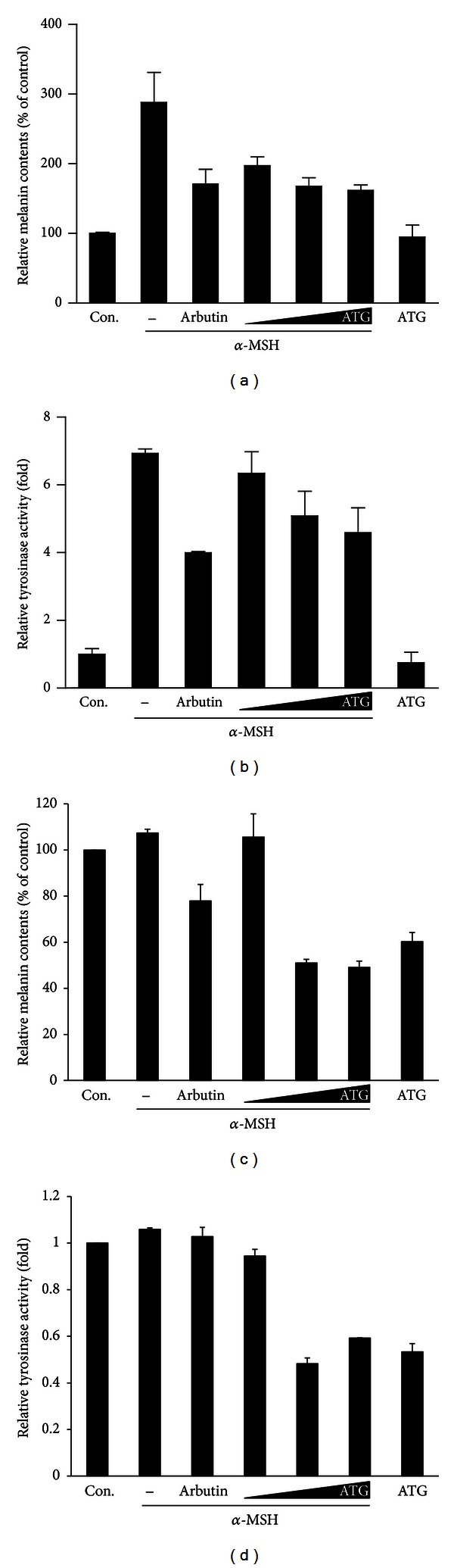
Effect of ATG (1, 10, and 50 *μ*M) on *α*-MSH (100 nM) stimulated murine B16BL6 melanoma cells ((a) and (b)) and melan-A cells ((c) and (d)). Data are represented as relative melanin contents and tyrosinase activity compared with arbutin (100 **μ**g/mL). ATG concentration without *α*-MSH was 10 *μ*M. All values were represented from three independent experiments with mean ± SD values.

**Figure 4:**
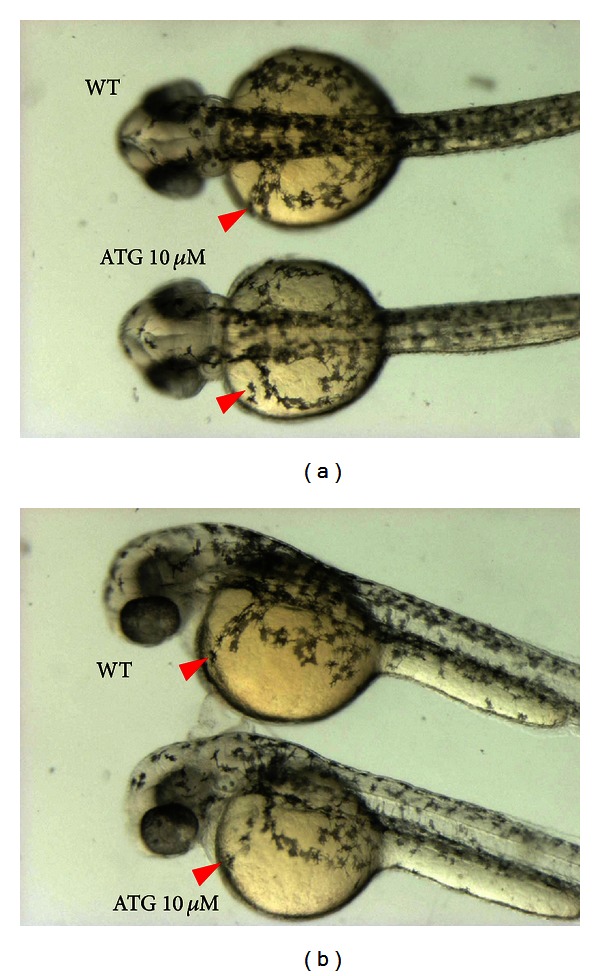
ATG treatment suppressed pigment deposition without affecting cell migration. Moderate concentration of ATG (10 *μ*M) treated embryos showed reduced embryonic pigmentation without causing any discernible pigment cells migration defect (red arrowhead). Treatment of high dose ATG (100 *μ*M) caused severe embryonic developmental retardation and growth was stalled at 18–20 somite stage (data not shown). All live embryos were treated with ATG for different dosages at 15 hpf (12–14 somite stage) followed by taken picture at 40 hpf. Pictures were shown by overview (a) and lateral view (b).

To further confirm that ATG is an inhibitory active compound, the effect of ATG on melanin content and tyrosinase activity was analyzed in B16BL6 melanoma cells and nonneoplastic melan-A cells. Cellular melanin contents and tyrosinase activities were significantly reduced by ATG treatment in a dose-dependent manner in both B16BL6 and melan-A cells, with similar inhibition pattern between two cell lines (Figures 3(a), 3(b), 3(c), and 3(d)). As shown in the result of melanin contents, ATG exhibited inhibitory activity on melanin contents similar to the positive control arbutin at the concentrations of 10 and 50 *μ*M in both B16BL6 and melan-A cells. The inhibitory effect of ATG on tyrosinase activity was also significant in both two cell lines at concentrations of 10 and 50 *μ*M similar with arbutin. Particularly in melan-A cells, inhibitory effect on melanin contents and tyrosinase activity was significantly stronger than that of arbutin control, at the concentrations of 10 and 50 *μ*M. For in vivo animal model, ATG treatment (10 *μ*M) prior to the emergence of pigmentation in 15 hpf developing zebrafish embryos showed the moderate reduction of pigment deposition (40%, *n* = 52, Figure 4). The embryos were normally developed without any discernible developmental retardation as well as morphological defects. However, 10-fold higher dose treatment of ATG became harmful to the embryos since the ATG treated (100 *μ*M) embryos did not undergo normal development but were instead stalled at between 18 and 20 somite stage (100%, *n* = 47, data not shown). Lower dose of ATG (1 *μ*M) failed to suppress the body pigmentation (100%, *n* = 54). Analysis of tyrosinase activity has been widely used as a measure of melanin synthesis in cell culture systems [41]. For this reason, measuring both tyrosinase activity and melanin contents is usually conducted when evaluating the melanogenesis inhibitory effect of natural products or certain compounds. Therefore, we measured the effect of ATG on *α*-MSH-mediated tyrosinase activity. Consistent with the results observed in the melanin content assay, ATG treatment significantly suppressed tyrosinase activity. Other natural herbs and medicines have been shown to inhibit tyrosinase activity and melanin contents. For example, Zhong et al. screened 90 traditional Chinese herb extracts to identify extracts displaying depigmentation activity [42]. Among these, the water extract of *Galla Chinensis* and ethanol extract of Radix Clematidis showed the highest activity in the cell culture assay. Although they did not identify the active compound in each extracts further investigations are needed for the development of useful dermatological agents. Lin et al. isolated biochanin A from the methanol extract of *Trifolium pretense* and found that it inhibited melanogenesis and tyrosinase activity [43]. They also confirmed its activity after dermatological application to mouse skin. In addition, 7-methoxycoumarin was isolated as an active compound for antimelanogenesis from the methanol extract of the leaves of *Eupatorium triplinerve* Vahl [44].

### 3.4. Regulatory Effect of ATG on Promoter Activity and cAMP Signaling

**Figure 5:**
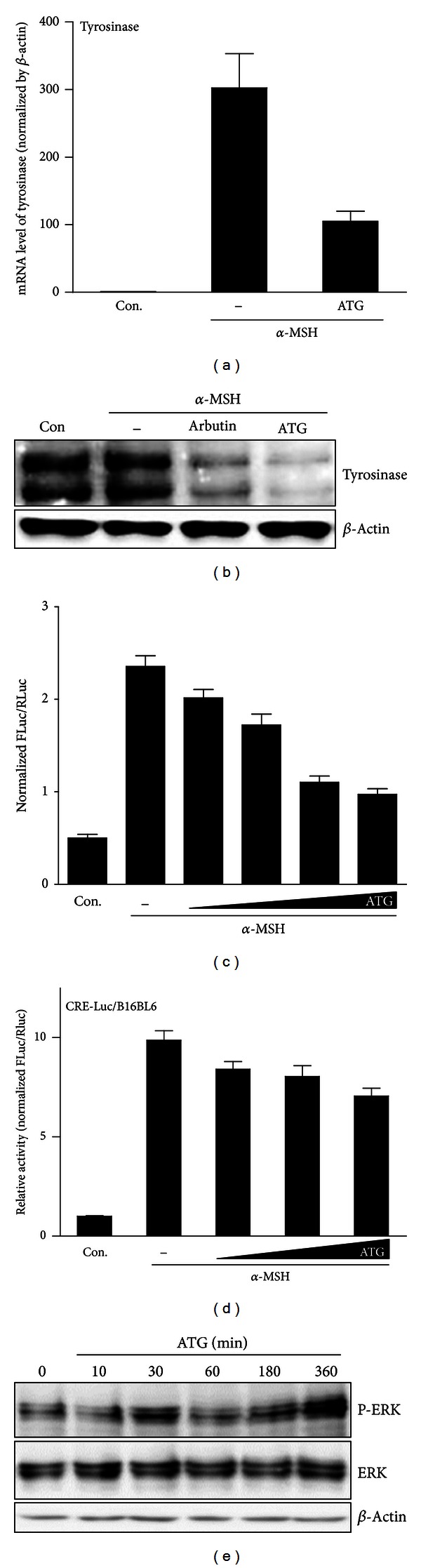
Inhibitory effect of ATG (10 *μ*M) on tyrosinase gene expression was analyzed by real-time qPCR (a) and western blot (b) in B16BL6 cells stimulated with *α*-MSH. The effect of ATG (1, 10, 20, and 50 *μ*M) on tyrosinase promoter activity was determined using the luciferase reporter system (c). Dose-dependent inhibitory effect of ATG (1, 10, and 50 *μ*M) was observed in the CRE-luciferase assay (d), and the level of P-ERK increased in a time-dependent manner (e).

Since ATG inhibited cellular melanin synthesis and tyrosinase activity, we examined whether ATG could affect the expression of tyrosinase and intracellular signaling pathway. ATG significantly inhibited *α*-MSH-induced tyrosinase mRNA expression and tyrosinase protein expression was also greatly repressed to levels greater than the arbutin control (Figures 5(a) and 5(b)). These results imply that ATG downregulates tyrosinase gene expression, which would decrease tyrosinase enzyme activity and ultimately inhibit melanogenesis. ATG may lower the tyrosinase activity by regulating posttranslational modification and degradation of tyrosinase protein in the cells. To confirm the effect of ATG on tyrosinase gene expression, we analyzed tyrosinase promoter activity using luciferase reporter assay, which encompasses the promoter region (−2267 to +19). As shown in Figure 5(c), we found that ATG reduced the promoter activity in a dose-dependent manner. This result demonstrated that this promoter region contains an important regulatory element that is involved in cAMP. In addition, it was reported that the tyrosinase mRNA level can be regulated by *α*-MSH and cAMP in mouse melanoma cells [45, 46]. They observed increased tyrosinase activity and mRNA expression and suggested that *α*-MSH promotes tyrosinase expression and activity by acting through cAMP.

Recently, Bertolotto et al. demonstrated that cAMP level was closely related with the transcriptional activity of tyrosinase gene [47]. They showed that an elevated cAMP level can be a strong stimulator of the tyrosinase promoter. They also reported that the M-box and E-box, which is located up- and downstream of the TATA-box ahead of the initiation site, are involved in the regulation of tyrosinase promoter activity by cAMP. The tyrosinase promoter region of DNA they analyzed was very similar to the region analyzed in this study. These results imply that ATG may inhibit tyrosinase activity and melanogenesis by altering gene expression through the formation of DNA-protein complexes and signaling pathways (especially with regard to cAMP).

Moreover, it is well known that *α*-MSH stimulates melanin synthesis and also acts as a cAMP-elevating agent, which indicates that cAMP plays an important role in the regulation of melanogenesis [48]. It was also shown that ERK activation (phosphorylation of ERK) can be induced by physical factors and natural compounds [49–51]. cAMP and ERK signaling are key regulatory elements in the pathways of cell proliferation and differentiation and also play an important role in melanogenesis [52]. In mammals, cAMP has an important function in pigmentation, where it was shown to increase the expression of MITF through the activation of protein kinase A, which in turn stimulates tyrosinase gene expression for melanin synthesis [53]. cAMP-elevating agents, such as *α*-MSH, IBMX, and forskolin, also stimulate melanin synthesis. Moreover, it is known that *α*-MSH, which is a signal transducer, potently induces MITF expression and increases melanin synthesis. cAMP-related cellular signaling and the molecular mechanisms involved in different biochemical pathways have been widely studied. cAMP increases the expression of MITF by activating PKA which phosphorylate its substrates, enzymes, and regulatory proteins, and then PKA phosphorylates cAMP response element binding protein (CREB). Tyrosinase gene expression and melanin synthesis are then stimulated by this signaling pathway, and *α*-MSH stimulates cAMP upregulation. This then induces melanogenesis, which is closely linked to the level of tyrosinase [54, 55]. To check whether ATG has an effect on this signaling pathway mainly involving cAMP, the CRE-Luc reporter assay was performed. In this analysis, ATG was shown to reduce the cAMP level in B16BL6 cells of reporter transfected (Figure 5(d)). Consequently, we found that ATG regulates tyrosine activity and inhibits melanogenesis by inhibiting cAMP levels and even further contributes in suppressing melanin synthesis.

In a previous study, acteoside from the leaves of *Rehmannia glutinosa* was shown to inhibit melanogenesis by ERK activation and tyrosinase downregulation [56]. According to this report, we determined if ATG can act as ERK activating agent in repressing melanin and tyrosinase synthesis. As shown in the Figure 5(e), the level of p-ERK increased with ATG (10 *μ*m) treatment time and the effect was remarkably after 30 min. This result is in agreement with previous reports on the ERK pathway in melanogenesis [57]. ATG exerts significant inhibitory activity on tyrosinase and melanin synthesis by blocking the cAMP pathway and activating the ERK pathway. Consequently, ATG significantly inhibit tyrosinase activity, expression, and melanogenesis.

In conclusion, we first investigated the inhibitory effect of ATG on tyrosinase activity and melanin biosynthesis and elucidated the signaling mechanism. We found ATG has a significant inhibitory effect on them without producing any significant cytotoxicity. Those results indicate that ATG inhibits melanogenesis in murine melanoma B16BL6 cells by repressing cAMP and tyrosinase gene/protein expression and increasing ERK phosphorylation in the signaling pathway of melanogenesis. Likewise, inhibition of melanogenesis was also confirmed in nonneoplastic cells melan-A and in vivo zebrafish embryos. However, ATG of high concentration 100 *μ*M was shown to have toxicity in zebrafish embryos with developmental retardation. The results of this study imply that ATG may be used as a natural resource for the treatment of melanogenesis, which is highly important given the current increase in demand for complementary and alternative medicines.

## References

[1] Cordell GA, Colvard MD (2012). Natural products and traditional medicine: turning on a paradigm. *Journal of Natural Products*.

[2] Kim H, Choi HR, Kim DS, Park KC (2012). Topical hypopigmenting agents for pigmentary disorders and their mechanisms of action. *Annals of Dermatology*.

[3] Solano F, Briganti S, Picardo M, Ghanem G (2006). Hypopigmenting agents: an updated review on biological, chemical and clinical aspects. *Pigment Cell Research*.

[4] Nguyen DTM, Nguyen DH, Lyun HL, Lee HB, Shin JH, Kim EK (2007). Inhibition of melanogenesis by dioctyl phthalate isolated from *Nigella glandulifera* Freyn. *Journal of Microbiology and Biotechnology*.

[5] Lv N, Koo JH, Yoon HY (2007). Effect of Angelica gigas extract on melanogenesis in B16 melanoma cells. *International Journal of Molecular Medicine*.

[6] Windisch W, Schedle K, Plitzner C, Kroismayr A (2008). Use of phytogenic products as feed additives for swine and poultry. *Journal of Animal science*.

[7] Mukherjee PK, Ponnusankar S, Pandit S, Hazam PK, Ahmmed M, Mukherjee K (2011). Botanicals as medicinal food and their effects on drug metabolizing enzymes. *Food and Chemical Toxicology*.

[8] McGovern PE, Christofidou-Solomidou M, Wang W, Dukes F, Davidson T, El-Deiry WS (2010). Anticancer activity of botanical compounds in ancient fermented beverages. *International Journal of Oncology*.

[9] Guimarães R, Barros L, Carvalho AM, Ferreira ICFR (2010). Studies on chemical constituents and bioactivity of *Rosa micrantha*: an alternative antioxidants source for food, pharmaceutical, or cosmetic applications. *Journal of Agricultural and Food Chemistry*.

[10] Shu YT, Weng CS, Kuo WG, Jiang GJ, Kao KT (2005). The investigation on the skin whitening efficacy of Chinese herb extracts using the colour physics technology. *Journal of Dermatological Science*.

[11] Park JH, Shin YG, Shin UK (1997). Tyrosinase inhibition activity of some herbal drugs. *Yakhak Hoeji*.

[12] Yang M, Kim M, Lim S, Ann H, Ahn R (1999). Inhibitory effects of water-acetone extracts of chestnut inner shell, pine needle and hop on the melanin biosynthesis. *Yakhak Hoeji*.

[13] Gillbro JM, Olsson MJ (2011). The melanogenesis and mechanisms of skin-lightening agents—existing and new approaches. *International Journal of Cosmetic Science*.

[14] Mapunya MB, Nikolova RV, Lall N (2012). Melanogenesis and antityrosinase activity of selected South African plants. *Evidence-Based Complementary and Alternative Medicine*.

[15] Sturm RA, Teasdale RD, Box NF (2001). Human pigmentation genes: identification, structure and consequences of polymorphic variation. *Gene*.

[16] Prota G (1980). Recent advances in the chemistry of melanogenesis in mammals. *Journal of Investigative Dermatology*.

[17] Korner A, Pawelek J (1982). Mammalian tyrosinase catalyzes three reactions in the biosynthesis of melanin. *Science*.

[18] Bertolotto C, Abbe P, Hemesath TJ (1998). Microphthalmia gene product as a signal transducer in cAMP-induced differentiation of melanocytes. *Journal of Cell Biology*.

[19] Price ER, Horstmann MA, Wells AG (1998). *α*-melanocyte-stimulating hormone signaling regulates expression of Microphthalmia, a gene deficient in Waardenburg syndrome. *The Journal of Biological Chemistry*.

[20] Le Pape E, Wakamatsu K, Ito S, Wolber R, Hearing VJ (2008). Regulation of eumelanin/pheomelanin synthesis and visible pigmentation in melanocytes by ligands of the melanocortin 1 receptor. *Pigment Cell and Melanoma Research*.

[21] Matsumoto T, Hosono-Nishiyama K, Yamada H (2006). Antiproliferative and apoptotic effects of butyrolactone lignans from *Arctium lappa* on leukemic cells. *Planta Medica*.

[22] Matsuzaki Y, Koyama M, Hitomi T (2008). Arctiin induces cell growth inhibition through the down-regulation of cyclin D1 expression. *Oncology Reports*.

[23] Knipping K, Van Esch ECAM, Wijering SC, Van Der Heide S, Dubois AE, Garssen J (2008). *In vitro* and *in vivo* anti-allergic effects of *Arctium lappa* L. *Experimental Biology and Medicine*.

[24] Knott A, Reuschlein K, Mielke H (2008). Natural *Arctium lappa* fruit extract improves the clinical signs of aging skin. *Journal of Cosmetic Dermatology*.

[25] Wu JG, Wu JZ, Sun LN (2009). Ameliorative effects of arctiin from *Arctium lappa* on experimental glomerulonephritis in rats. *Phytomedicine*.

[26] Moritani S, Nomura M, Takeda Y, Miyamoto KI (1996). Cytotoxic components of bardanae fructus (goboshi). *Biological and Pharmaceutical Bulletin*.

[27] Takasaki M, Konoshima T, Komatsu K, Tokuda H, Nishino H (2000). Anti-tumor-promoting activity of lignans from the aerial part of *Saussurea medusa*. *Cancer Letters*.

[28] Bastos MMSM, Kijjoa A, Cardoso JM, Gutierrez Herz ABW (1990). Lignans and other constituents of *Centaurea sphaerocephala* ssp. *Polyacantha*. *Planta Medica*.

[29] Liu H, Zhang Y, Sun Y (2010). Determination of the major constituents in fruit of Arctium lappa L. by matrix solid-phase dispersion extraction coupled with HPLC separation and fluorescence detection. *Journal of Chromatography B*.

[30] Jang JY, Lee JH, Kang BW, Chung KT, Chol YH, Choi BT (2009). Dichloromethane fraction of *Cimicifuga heracleifolia* decreases the level of melanin synthesis by activating the ERK or AKT signaling pathway in B16F10 cells. *Experimental Dermatology*.

[31] Wang HM, Chen CY, Wen ZH (2011). Identifying melanogenesis inhibitors from *Cinnamomum subavenium* with *in vitro* and *in vivo* screening systems by targeting the human tyrosinase. *Experimental Dermatology*.

[32] Kim DS, Kim SY, Park SH (2005). Inhibitory effects of 4-n-butylresorcinol on tyrosinase activity and melanin synthesis. *Biological and Pharmaceutical Bulletin*.

[33] Lin YP, Hsu FL, Chen CS, Chern JW, Lee MH (2007). Constituents from the Formosan apple reduce tyrosinase activity in human epidermal melanocytes. *Phytochemistry*.

[34] Choi TY, Kim JH, Ko DH (2007). Zebrafish as a new model for phenotype-based screening of melanogenic regulatory compounds. *Pigment Cell Research*.

[35] Chan YS, Cheng LN, Wu JH (2011). A review of the pharmacological effects of *Arctium lappa* (burdock). *Inflammopharmacology*.

[36] Yang S, Ma J, Xiao J (2012). Arctigenin anti-tumor activity in bladder cancer t24 cell line through induction of cell-cycle arrest and apoptosis. *The Anatomical Record*.

[37] Awale S, Lu J, Kalauni SK (2006). Identification of arctigenin as an antitumor agent having the ability to eliminate the tolerance of cancer cells to nutrient starvation. *Cancer Research*.

[38] Hayashi K, Narutaki K, Nagaoka Y, Hayashi T, Uesato S (2010). Therapeutic effect of arctiin and arctigenin in immunocompetent and immunocompromised mice infected with influenza a virus. *Biological and Pharmaceutical Bulletin*.

[39] Kim JH, Bae JT, Song MH, Lee GS, Choe SY, Pyo HB (2010). Biological activities of fructus arctii fermented with the basidiomycete *Grifola frondosa*. *Archives of Pharmacal Research*.

[40] Zhao F, Wang L, Liu K (2009). In vitro anti-inflammatory effects of arctigenin, a lignan from *Arctium lappa* L., through inhibition on iNOS pathway. *Journal of Ethnopharmacology*.

[41] Hu DN (2008). Methodology for evaluation of melanin content and production of pigment cells *in vitro*. *Photochemistry and Photobiology*.

[42] Zhong S, Wu Y, Soo-Mi A (2006). depigmentation of melanocytes by the treatment of extracts from traditional Chinese herbs: a cell culture assay. *Biological and Pharmaceutical Bulletin*.

[43] Lin VC, Ding HY, Tsai PC, Wu JY, Lu YH, Chang TS (2011). *In Vitro* and *in Vivo* melanogenesis inhibition by biochanin a from *Trifolium pratense*. *Bioscience, Biotechnology and Biochemistry*.

[44] Arung ET, Kuspradini H, Kusuma IW, Shimizu K, Kondo R (2012). Validation of *Eupatorium triplinerve* Vahl leaves, a skin care herb from East Kalimantan, using a melanin biosynthesis assay. *Journal of Acupunctute and Meridian Studies*.

[45] Hoganson GE, Ledwitz-Rigby F, Davidson RL, Fuller BB (1989). Regulation of tyrosinase mRNA levels in mouse melanoma cell clones by melanocyte-stimulating hormone and cyclic AMP. *Somatic Cell and Molecular Genetics*.

[46] Rungta D, Corn TD, Fuller BB (1996). Regulation of tyrosinase mRNA in mouse melanoma cells by *α*-melanocyte-stimulating hormone. *Journal of Investigative Dermatology*.

[47] Bertolotto C, Abbe P, Hemesath TJ (1998). Microphthalmia gene product as a signal transducer in cAMP-induced differentiation of melanocytes. *Journal of Cell Biology*.

[48] Hunt G, Todd C, Cresswell JE, Thody AJ (1994). *α*-Melanocyte stimulating hormone and its analogue Nle4DPhe7*α*-MSH affect morphology, tyrosinase activity and melanogenesis in cultured human melanocytes. *Journal of Cell Science*.

[49] Yanase H, Ando H, Horikawa M, Watanabe M, Mori T, Matsuda N (2001). Possible involvement of ERK 1/2 in UVA-induced melanogenesis in cultured normal human epidermal melanocytes. *Pigment Cell Research*.

[50] Kim DS, Park SH, Kwon SB (2006). Sphingosylphosphorylcholine-induced ERK activation inhibits melanin synthesis in human melanocytes. *Pigment Cell Research*.

[51] Kawano M, Matsuyama K, Miyamae Y (2007). Antimelanogenesis effect of Tunisian herb *Thymelaea hirsuta* extract on B16 murine melanoma cells. *Experimental Dermatology*.

[52] Buscà R, Ballotti R (2000). Cyclic AMP a key messenger in the regulation of skin pigmentation. *Pigment Cell Research*.

[53] Schallreuter KU, Kothari S, Chavan B, Spencer JD (2008). Regulation of melanogenesis-controversies and new concepts. *Experimental Dermatology*.

[54] Englaro W, Rezzonico R, Durand-Clement M, Lallemand D, Ortonne - JP, Ballotti R (1995). Mitogen-activated protein kinase pathway and AP-1 are activated during cAMP-induced melanogenesis in B-16 melanoma cells. *The Journal of Biological Chemistry*.

[55] Steingrímsson E, Copeland NG, Jenkins NA (2004). Melanocytes and the Microphthalmia transcription factor network. *Annual Review of Genetics*.

[56] Son YO, Lee SA, Kim SS, Jang YS, Chun JC, Lee JC (2011). Acteoside inhibits melanogenesis in B16F10 cells through ERK activation and tyrosinase down-regulation. *Journal of Pharmacy and Pharmacology*.

[57] Li X, Guo L, Sun Y, Zhou J, Gu Y, Li Y (2010). Baicalein inhibits melanogenesis through activation of the ERK signaling pathway. *International Journal of Molecular Medicine*.

